# Comparison of Milk Removal Efficiency and Maternal Perception Between a Hospital-Grade and Personal-Use Pump

**DOI:** 10.3390/healthcare14152251

**Published:** 2026-07-23

**Authors:** Ashleigh H. Warden, Zoya Gridneva, Jacki L. McEachran, Sarah G. Abelha, Sharon L. Perrella, Donna T. Geddes

**Affiliations:** 1 School of Molecular Sciences, The University of Western Australia, Crawley, WA 6009, Australia; ashleigh.warden@uwa.edu.au (A.H.W.); jacki.mceachran@uwa.edu.au (J.L.M.); sarah.abelha@uwa.edu.au (S.G.A.); sharon.perrella@uwa.edu.au (S.L.P.); donna.geddes@uwa.edu.au (D.T.G.); 2 ABREAST Network, Perth, WA 6000, Australia; 3 UWA Centre for Human Lactation Research and Translation, Crawley, WA 6009, Australia

**Keywords:** electric breast pump, breast expression, vacuum, breastfeeding, lactation, milk expression, hospital-grade pump, personal-use pump, comfort, maternal satisfaction

## Abstract

**Highlights:**

**What are the main findings?**

- Hospital-grade pump (Symphony) and personal-use pump (Pump in Style Pro) performance did not show differences in milk removal efficacy and comfort.
- Participants perceived meaningful differences in pump experience, rating the hospital-grade pump’s vacuum as smoother and its sound as more pleasant than the personal-use pump.

**What are the implications of the main findings?**

- The absence of difference in milk removal efficacy with the personal-use pump (Pump in Style Pro) suggests it may be a viable and more accessible alternative for women when transitioning from a hospital-grade pump.
- Subjective perceptions of vacuum smoothness and pleasantness of sound even in the absence of performance differences may influence maternal pump preference and satisfaction, highlighting the importance of considering user experience alongside efficacy when guiding pump selection.

**Abstract:**

**Background/Objectives**: Breast pumps are essential tools when breastfeeding dyads are separated or there are breastfeeding challenges that prevent or reduce effective milk removal. Hospital-grade and personal-use pumps offer different features and performance characteristics. Direct comparative studies evaluating pump efficacy and maternal satisfaction are limited in this area, hampering healthcare professionals in their support of lactating women. We compared the efficacy and maternal perceptions of a hospital-grade breast pump (Symphony; Medela AG) and an electric personal-use pump (Pump in Style Pro; Medela AG). **Methods**: In total, 28 lactating mothers with healthy-term infants participated in an unblinded randomised crossover trial. Participants attended two pumping sessions using either the hospital-grade pump or personal-use pump. Effectiveness of milk removal and maternal perception were measured. Participants completed a 24 h milk profile to enable calculation of the percentage of available milk removed from the breast. **Results**: There were no significant differences for milk removal efficacy parameters after accounting for pre-expression degree of fullness of the breast between pumps. Similarly, there was no difference in comfort ratings. However, mothers perceived the hospital-grade breast pump vacuum as smoother than the personal-use pump (*p* = 0.003) and rated the hospital-grade breast pump as having a more pleasant sound (*p* < 0.001). Both findings remained significant after adjustment for pre-expression degree of fullness (*p* < 0.001 and *p* = 0.030, respectively). **Conclusions**: The hospital-grade pump and the personal-use pump did not differ with respect to milk removal efficacy and comfort. However, participants did perceive differences in vacuum application.

## 1. Introduction

Regular milk expression with an effective breast pump is a key strategy in clinical lactation management where direct breastfeeding is compromised or interrupted. In circumstances including preterm birth, infant hospitalisation, maternal–infant separation, and lactation pathologies such as insufficient milk production [1] or ineffective infant attachment, breast pumps are used to stimulate and maintain mammary gland secretory activity whilst ensuring the continued provision of human milk for the infant [2,3,4,5].

Currently, there is a dearth of evidence-based information available to health professionals with respect to establishment and maintenance of lactation when breastfeeding is not possible [5,6]. In these cases, there is a strong clinical need for an effective method of milk removal, as the stimulation of the breast, in addition to effective milk removal, is critical to secretory activation [7,8] and autocrine control of milk synthesis [9,10]. Whilst electric breast pumps have been found to be more efficacious compared to hand expression [11,12,13,14,15,16], typically no evaluation of effectiveness or comfort [17] is conducted and/or widely available for these devices [18,19,20]. Both parents and healthcare professionals require evidence-based guidelines in order to individualise the choice and use of the breast pumps to support lactation [21]. The need for transparent standardised efficacy evaluation of breast pumps has been flagged consistently by health professionals [9], yet most studies in the published literature usually refer to volume of milk removed, without controlling for potential confounders or evaluating milk removal rate [22,23].

The expanding utilisation of breast pumps has driven the development of two distinct device categories: hospital-grade (multiuser) pumps and personal-use (single-user) pumps [24]. Hospital-grade breast pumps are considered the clinical standard of care in neonatal intensive care units and tertiary lactation services [9]. These devices are engineered to deliver consistent, evidence-based vacuum patterns that facilitate the initiation and maintenance of milk synthesis, particularly among at-risk populations and feature a “closed system” to ensure hygienic, multiuser capability. Personal-use pumps, however, are highly variable in terms of technology and shield design, resulting in perceived differences in efficacy compared to hospital-grade pumps [21]. Furthermore, maternal acceptability is an important consideration where adherence to frequent milk expression is required [25,26].

Direct comparative investigations of breast pumps evaluating their milk removal efficacy and maternal perceptions remain scarce [27]. The existing evidence base is largely confined to studies examining individual pump performance in isolation [13,15] or comparing mechanical expression against direct breastfeeding [11].

This exploratory study was designed to directly compare both the milk removal efficacy and maternal perceptions of comfort associated with a hospital-grade breast pump (Symphony; Medela AG) and an electric personal-use breast pump (Pump in Style Pro; Medela AG).

## 2. Materials and Methods

### 2.1. Study Design

**Figure 1:**
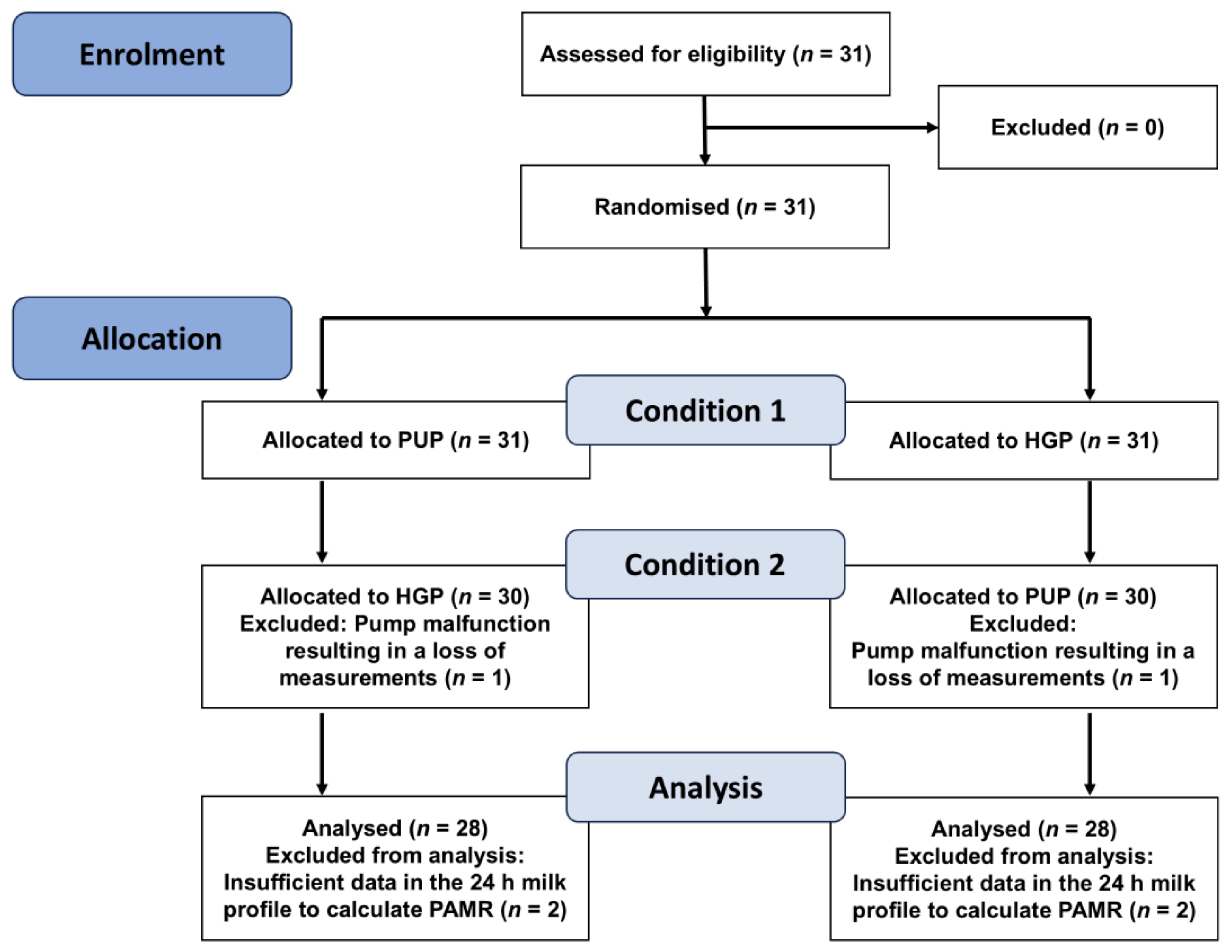
Study design. HGP, hospital-grade pump (Symphony (Medela AG)); PAMR, percentage of available milk removed; PUP, personal-use pump (Pump in Style Pro (Medela AG)).

This study employed a randomised crossover design (Figure 1) in which participants attended two separate sessions at The University of Western Australia. At each session, participants expressed milk using either a hospital-grade pump (HGP; Symphony hospital-grade breast pump (Medela AG, Baar, Switzerland)) or an electric personal-use pump (PUP; Pump in Style Pro (Medela AG, Baar, Switzerland)). The order of pump allocation was randomised in blocks of 10 using R statistical software (version 4.4.2; R Foundation for Statistical Computing, Vienna, Austria) to ensure balanced assignment throughout the study period. Blinding was not possible due to the differences in pump size, sound and connection kit configuration. Each pump was tested on a separate day, with participants returning for a subsequent visit (3 (3.5) weeks apart) to express with the alternate device. Each PUP session used a new pump. At each session, participants double-pumped for 15 min. All equipment was sterilised prior to use. The primary outcome was effectiveness of milk removal. Secondary outcomes included milk flow dynamics and maternal perceptions of pump performance.

### 2.2. Participants

Lactating mothers between 1 and 6 months postpartum were recruited for this study. Ethical approval was obtained from the Human Research Ethics Committee of The University of Western Australia (RA/4/20/6407). This study was conducted in accordance with the Declaration of Helsinki and all participants provided written informed consent prior to enrolment.

Eligible participants were English-speaking mothers aged 18 years or older of healthy-term infants, who were predominantly breastfeeding their infant and had experience of using a breast pump. Participants were excluded if they were predominantly feeding commercial milk formula, practicing tandem feeding, or had given birth to twins or higher-order multiples. Participant demographics and lactation characteristics were collected via an online questionnaire administered prior to the first study session.

### 2.3. Pumping Sessions

#### 2.3.1. Nipple Measurements and Expression Protocol

Nipple temperature was recorded both pre- and post-expression using a FLIR T650sc thermal camera (thermal sensitivity < 20 mK @ 30 °C; accuracy 0.02 °C; FLIR Systems Inc., Wilsonville, OR, USA). Pre-expression temperatures were obtained following a brief equilibration period (~1.5 min). Small milk samples (<1 mL) were collected by manual expression into 5 mL polypropylene tubes (P5016SL, Techno Plas Pty Ltd., St. Marys, SA, Australia) before and after pumping.

Based on pre-expression nipple diameter measurements with an electronic digital vernier calliper (Performance Tool, Renton, WA, USA) to the closest 0.1 mm, participants were fitted with a 21 or 24 mm PersonalFit™ PLUS Breast Shield (Medela AG, Baar, Switzerland), selected to provide a shield diameter approximately 4 mm wider than the nipple base. The shield was positioned so the nipple was centred within the tunnel. Participants selected the vacuum level, so it was set at the maximum comfortable vacuum for both stimulation and expression phases. Both pumps were connected to a customised computer that recorded the vacuum waveform and strength continuously throughout the expression session. Participants expressed for 15 min from the onset of milk ejection. Milk volume and milk flow rates were measured for the left breast using the ShowMilk device (Medela AG, Baar, Switzerland) [28,29], with milk flow rate (g/s) calculated as the derivative of weight change over time. Once milk ejection was detected, the vacuum pattern was switched from the stimulation phase to the expression phase. If milk ejection had not occurred within 1 min, the pump switched automatically to the expression phase.

At the conclusion of the pumping session, the nipple was gently dried to remove residual milk immediately prior to recording post-expression temperature.

#### 2.3.2. Vacuum Curves

**Figure 2:**
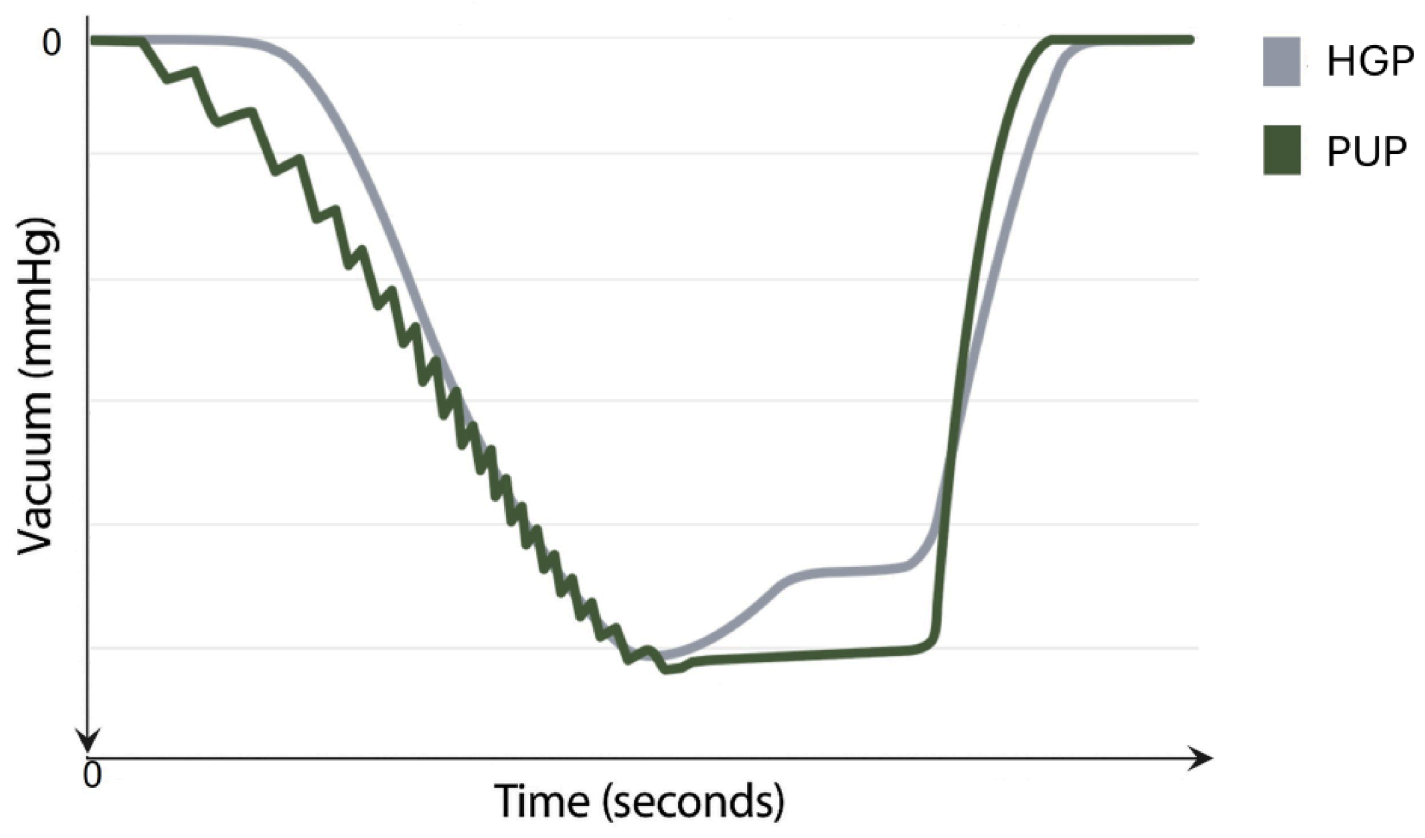
Comparison of the vacuum curves applied during the expression phase of the MAINTAIN programme for the HGP (hospital-grade pump; Symphony (Medela AG)) and PUP (personal-use pump; Pump in Style Pro (Medela AG)).

Both the HGP and the PUP employ Medela’s 2-Phase Expression^®^ technology (Medela AG, Baar, Switzerland), which comprises a high-frequency stimulation phase designed to elicit milk ejection followed by a slower expression phase optimised for milk removal. The HGP generates its vacuum waveform via a precision piston-driven motor system capable of delivering a smooth, sinusoidal-like vacuum curve characterised by a gradual rise to peak negative pressure, a rounded hold at the apex, and a gentle, progressive release back to baseline across each cycle. This produces a waveform contour with smooth transitions between phases of the cycle and minimal abrupt inflection points. The HGP curve operates with a stimulation phase of 120 cycles/min, transitioning to an expression phase of 54–78 cycles/min, with a vacuum range of −50 to −250 mmHg. The PUP was engineered to replicate the HGP vacuum pattern, featuring the same 16 adjustable vacuum levels across both stimulation and expression phases and a comparable vacuum range. However, inherent differences in motor size, drive mechanism, and engineering constraints between a multiuser hospital-grade device and a compact PUP result in measurable differences in the vacuum waveform geometry (Figure 2). The PUP produces a more trapezoidal waveform profile, with a steeper rise to peak vacuum, a flatter and less rounded hold phase, and a more rapid release back to baseline, resulting in sharper transitions between the build, hold, and release components of each cycle. These angular inflection points contrast with the HGP’s characteristically rounded waveform and may contribute to the perceptual differences.

### 2.4. Determination of Percentage of Available Milk Removed

To calculate the percentage of available milk removed (PAMR) during pumping sessions, participants completed a 24 h milk production profile at their home [30]. Participants recorded weight for each feed or expression electronically and collected small milk samples; these data, together with pre- and post-feed/expression milk fat concentrations, were used to calculate the degree of fullness of the breast pre- and post-expression, breast storage capacity, available milk, and PAMR of the pumping sessions, following previously described methods [31,32,33,34].

### 2.5. User Experience

Participants completed a user experience questionnaire during and after pumping. Questions relating to maternal comfort, vacuum and sound perception were asked during pumping. Questions relating to satisfaction and comparisons to the mother’s home pumps were asked after the session was completed. Participants rated their experience on a 5-point Likert scale, with 1 being the most negative response and 5 being the most positive response.

### 2.6. Statistical Analysis

All analyses were conducted using R statistical software (version 4.4.2; R Foundation for Statistical Computing, Vienna, Austria). An a priori sample size calculation was performed in R using the power.t.test function for a paired, one-sided non-inferiority design. Assuming a non-inferiority margin of 6.5 PAMR points, a standard deviation of the within-subject differences of 12.5, a significance level (*α*) of 0.05, and 80% power, a total of 25 participants were required, not accounting for dropouts. The sample size of 10–30 participants is considered sufficient for a pre-market study by the Therapeutic Goods Administration’s Australian clinical trial guidelines for medical devices [35].

Whilst we employed a randomised crossover design to minimise the between-subject variability, the sequence, period, and carry-over effects were not formally tested. Linear mixed-effects models were fitted using the lme function from the nlme package to compare outcomes between the two breast pumps. For each outcome variable, a linear mixed-effects model was specified with pump type as the fixed effect and participant as a random effect (random intercept model). To account for the potential confounding effects of baseline breast fullness on pump performance, adjusted models were fitted that included pre-expression degree of fullness as a covariate in the fixed-effects specification.

Outcome variables included milk volume measures (combined volume and left and right breast volumes separately), milk composition parameters (post-expression cream percentage and degree of fullness), milk ejection characteristics (time to first milk ejection and number of milk ejections), flow dynamics (overall flow duration, active flow duration, constant flow duration, milk removal rates, maximum flow rate, time to remove 80% and 90% of milk, time to stop pumping (time after which 0–<10% of milk is removed) and PAMR to time to stop), and maternal perceptions (sound pleasantness, comfort, satisfaction ratings, and vacuum characteristics including perceived strength, smoothness, and gentleness) [2].

Missing values for specific outcomes were excluded from the analysis of those variables on a per-variable basis. Statistical significance was set at *α* = 0.05 using two-tailed tests. To account for multiple comparisons and control the false discovery rate, we applied the Benjamini–Hochberg procedure. Adjusted *q*-values < 0.05 were considered statistically significant. Model coefficients, standard errors, degrees of freedom, *t*-values, *p*-values and *q*-values were extracted from the fixed effects output for each model. Both unadjusted and adjusted *p*-values and *q*-values are reported.

## 3. Results

### 3.1. Participants

Of the 31 participants enrolled, three were excluded from the final analysis: one due to pump malfunction during a session and two due to insufficient data recorded on the 24 h milk profiles, which precluded valid calculation of PAMR. Consequently, all analyses were based on data from the remaining 28 participants who completed both pumping sessions. Maternal and infant characteristics are presented in Table 1.

### 3.2. Milk Removal Parameters

The milk volumes and PAMR obtained with each pump are shown in Table 2. The HGP yielded greater combined milk volume compared to the PUP (147.1 ± 78.1 vs. 125.0 ± 73.5 g, *p* = 0.048); however, this difference was not significant after adjusting for pre-expression degrees of fullness (*p* = 0.84). The unadjusted difference was driven primarily by the left breast, where the HGP extracted more milk (75.3 ± 44.9 vs. 57.5 ± 45.8 g, *p* = 0.019), although this was non-significant after adjustment for breast fullness (*p* = 0.66). Right breast volumes did not differ significantly between pumps in either unadjusted or adjusted analyses. None of the differences were significant after the Benjamini–Hochberg procedure (Table 2).

Milk composition parameters, including post-expression cream percentage (14.4 ± 5.1 vs. 14.9 ± 4.58%, *p* = 0.91) and post-expression degree of fullness (0.08 ± 0.10 vs. 0.11 ± 0.14, *p* = 0.91), did not differ between pumps (Table 2). Further, PAMR was not significantly different between the HGP and PUP for the left breast (73.6 ± 17.2 vs. 68.4 ± 26.9%; *p* = 0.48), right breast (80.9 ± 23.4 vs. 81.1 ± 28.2%; *p* = 0.98), or both breasts combined (77.1 ± 20.4 vs. 74.7 ± 28.0%; *p* = 0.48). None of the differences were significant after the Benjamini–Hochberg procedure (Table 2).

Maximum comfortable vacuum did not differ between the pumps with respect to the stimulation vacuums (−120.0 ± 37.2 mmHg vs. −114.8 ± 34.7 mmHg, *p* = 0.45) and expression vacuums (−189.7 ± 53.8 mmHg vs. −177.8 ± 52.4 mmHg, *p* = 0.43). None of the differences were significant after the Benjamini–Hochberg procedure (Table 2).

### 3.3. Milk Ejection and Flow Characteristics

Milk ejection and flow parameters are presented in Table 3. The HGP demonstrated a higher constant flow rate (7.2 ± 3.6 vs. 5.4 ± 3.0 g/min, *p* = 0.004; *q* = 0.041), faster milk removal rate (4.9 ± 2.9 vs. 4.2 ± 2.8 g/min, *p* = 0.047), greater milk removal per minute of active flow duration (6.2 ± 3.1 vs. 4.8 ± 3.2 g/min, *p* = 0.012) and faster removal of 90% of total milk (10.2 ± 3.1 vs. 11.4 ± 3.3 min, *p* = 0.012). However, neither of these differences remained significant after adjusting for pre-expression breast fullness (*p* = 0.11, *q* = 0.82; *p* = 0.45; *p* = 0.33 and *p* = 0.27, respectively).

Other flow characteristics, including nipple temperature change, overall and active flow durations, maximum flow rate, and time to 80% milk removal did not differ significantly between pumps (*p* > 0.05 for all comparisons). None of the differences were significant after the Benjamini–Hochberg procedure (Table 3).

### 3.4. Maternal Perceptions

Maternal perceptions of pump characteristics are shown in Table 4. Participants rated the HGP as having a more pleasant sound than the PUP (4.6 ± 0.7 vs. 3.2 ± 0.8, *p* < 0.001; *q* < 0.001) and perceived its vacuum to be smoother (4.7 ± 0.5 vs. 3.9 ± 1.4, *p* = 0.003; *q* = 0.041), with both findings remaining significant after adjusting for pre-expression breast fullness (*p* < 0.001 and *p* = 0.030, respectively), with pleasantness of sound also remaining significantly different after the Benjamini–Hochberg procedure (*q* < 0.001).

The HGP also received better ratings compared to participants’ home pumps in the unadjusted analysis (3.8 ± 0.8 vs. 3.4 ± 0.8, *p* = 0.039), although this difference was not significant after adjustment for pre-expression breast fullness (*p* = 0.12). No significant differences were observed between pumps for maternal satisfaction with comfort, milk volume, or vacuum strength, or for perceptions of vacuum strength, comfort, or gentleness (*p* > 0.05 for all comparisons). None of the differences were significant after the Benjamini–Hochberg procedure (Table 4).

## 4. Discussion

This exploratory study demonstrated that there were no significant differences between the HGP (Symphony; Medela AG) and the PUP (Pump In Style Pro; Medela AG) with respect to milk removal efficacy, as measured by both expressed volume and PAMR during a 15 min simultaneous (double) expression session. Despite this absence of functional difference, participants were able to perceive differences in both vacuum application characteristics and the acoustic output of the two devices.

The total volume of milk removed during simultaneous expression with the HGP was greater than that obtained with the PUP (Table 2). However, after adjustment for the initial volume of milk available in the breast at the commencement of the session, this difference was no longer statistically significant. This finding underscores that expressed volume alone does not necessarily reflect the efficiency of milk removal relative to the volume available within the breast. For example, two women may each express 50 mL; however, if one had 50 mL available and the other 100 mL (a fuller breast), the former would have removed 100% of her available milk whilst the latter removed only 50%. This distinction is clinically important, as many previous breast pump and breast shield studies have interpreted differences in expressed milk volume as attributable to pump performance without controlling for baseline breast fullness, thereby limiting the validity of these conclusions. Furthermore, many studies have not controlled for the strength of vacuum applied, which is known to significantly influence milk removal efficacy [36,37]. Whilst participants in the present study selected their maximum comfortable vacuum at each session, this did not differ significantly between sessions, indicating that the differences in vacuum waveform between the two devices did not adversely impact milk removal and maternal comfort.

PAMR represents a more robust and physiologically relevant measure of milk removal efficacy, as it accounts for individual variations in milk availability at the time of expression [38,39]. In contrast to the differences observed in absolute volume expressed (before adjustment for degree of fullness), no significant difference in PAMR was identified between the two breast pumps, indicating that both devices removed milk from the breast with comparable efficacy. This finding is further supported by the absence of a significant difference in the change in nipple temperature between pumps, suggesting that neither the vacuum level nor the vacuum curve characteristics, which were themselves similar, adversely affected blood flow to the nipple during expression (Table 3). Moreover, the magnitude of change in nipple temperature observed with both devices was consistent with values previously documented for expression with the HGP [40,41].

Comfort during breast pump expression is an important clinical consideration, as pain and stress have been shown to partially inhibit the milk ejection reflex, resulting in diminished milk removal [42,43,44]. No significant difference in comfort ratings was observed between the HGP and the PUP, and on average, both pumps were rated as more comfortable than the participants’ own home pump (Table 4). The use of pumps with similar technology has previously been reported as ‘comfortable’ (level 4 or comparable to it) in a pre-market study of the Freestyle Hands-Free Breast Pump (Medela AG, Baar, Switzerland) [45] and in the study of experimental expression vacuum patterns [46].

Given that the vacuum curves of the two devices, while similar, are not identical, we investigated whether participants were able to perceive any differences in vacuum application. Ratings for perceived strength, comfort, and gentleness of the vacuum were not different between devices. However, participants rated the HGP as smoother than the PUP, indicating that lactating women are sensitive to subtle differences in the vacuum curve characteristics implemented in breast pumps, which may impact their experience [47,48,49].

Comfort is also influenced by other factors during pumping, such as shield fit, movement of the breast in the shield as the breast empties, pressure used to hold the shield (compression of breast tissue), and other factors such as audible noise from the pump. In this study, participants rated the HGP as having a more pleasant sound than the PUP (Table 4), and this difference remained significant after accounting for degree of fullness. Sound pleasantness during pumping may not be integral to effective milk removal but is an important aspect of comfort. Sound perception by participants is usually not reported by lactation studies that focus on breast expression with pumps. However, sound is part of multisensory stimuli and could induce an emotional response [50], which may further result in oxytocin production via the action of oxytocinergic nerves originating in the paraventricular nucleus [51], increasing blood flow to the nipple during breastfeeding/milk removal [52]. Sensory distractions, such as loud and sudden or jarring noises, may impede the milk ejection reflex, triggering cortisol release and a mild stress response, further inhibiting oxytocin release and milk removal [44,53], whilst music-based listening interventions have been reported to result in more milk removed during pumping sessions [54]. Nevertheless, the impact of vacuum or sound perception on long-term adherence to pumping remains speculative and requires prospective evaluation.

Efficiency of milk removal was not different between the two breast pumps, with no significant differences observed in latency to first milk ejection or the number of milk ejections, suggesting a similar physiological response to the vacuum applied to the breast by both devices [55]. Furthermore, the time to 80% and 90% of milk removed in addition to the time when pumping could have stopped suggested that pumping is not lengthened with the PUP. The absence of difference in milk removal efficacy between the pumps is most likely attributable to the close similarity of both the stimulation and expression vacuum curves for each pump. Whilst unadjusted analyses suggested that the HGP removed milk at a higher rate during active flow and reached 90% of total milk removed more rapidly, these differences did not persist in the adjusted models, indicating that greater milk availability in the breast at the HGP session accounted for the observed higher flow rates rather than any inherent difference in pump performance.

The demonstrated absence of a difference in milk removal efficacy between the HGP and the PUP, coupled with the shared 2-Phase Expression^®^ technology and comparable vacuum parameters, suggests that personal-use pumps with closely matched vacuum curves may offer a viable option for women transitioning from a HGP to a PUP. During the establishment period of lactation, when mothers of preterm or medically complex infants are frequently reliant on a HGP to initiate and build milk production, the transition to a personal-use device represents a critical juncture at which pump performance must be maintained to safeguard continued milk synthesis [9,56]. The present findings indicate that, at least under the controlled conditions of this study and in healthy-term breastfeeding mothers during established lactation who are experienced with pump use, milk removal efficacy was not compromised when participants expressed with a personal-use device employing a vacuum curve closely modelled on the hospital-grade standard. The comparable comfort ratings and the absence of any adverse effect on nipple blood flow also suggest that the expression experience was not detrimentally affected. Further studies in clinically pump-dependent women (e.g., mothers of preterm or ill infants) may be needed to confirm these findings in broader populations.

The strength of this study is its randomised crossover design, where each participant acts as their own control, minimising interindividual variability. Further, we used PAMR as a main effectiveness outcome rather than absolute milk volume removed and accounted for the pre-expression degree of breast fullness, which is a major confounding factor in lactation studies. We included subjective maternal perceptions of comfort, sound and vacuum smoothness, which may add clinical value and understanding to the importance of user-centred outcomes in breastfeeding support.

However, our exploratory study also has some limitations. As the sample size is relatively small, this study has limited statistical power and generalisability. Our sample consists of healthy breastfeeding mothers of term infants who were already experienced with breast pump use. Therefore, the conclusions may not be generalisable to mothers of preterm and/or medically fragile infants, pump-dependent mothers, those with lactation difficulties, or first-time pump users and those initiating lactation. Our study was unblinded, which could also have influenced maternal perception outcomes, especially regarding acoustic output, perceived vacuum smoothness, or comfort if participants had prior familiarity with hospital-grade devices. Further, the sequence, period, and carry-over effects were not formally tested. This was a cross-sectional study; thus, we could not investigate if there were any longitudinal differences regarding lactation outcomes such as milk supply, nipple trauma, or maternal fatigue, all of which are relevant in pump-dependent populations. Future longitudinal studies may be beneficial to inform breast pumping protocols and guidelines.

Additionally, as both breast pumps were produced by the same manufacturer and employ Medela’s 2-Phase Expression^®^ technology for vacuum generation, the findings could not be generalised to personal-use pumps from other manufacturers. Finally, this study was funded by an unrestricted research grant from Medela AG (Switzerland). Whilst Medela AG supplied the pumps for use in this evaluation, the funder had no role in the design of the study; collection, analyses, or interpretation of the data; writing of the manuscript; or decision to publish the results. The content is solely the responsibility of the authors and does not represent the official views of the funding body.

## 5. Conclusions

The hospital-grade breast pump (Symphony; Medela AG) and personal-use pump (Pump in Style Pro; Medela AG) demonstrated no difference in milk removal efficacy, as measured by the percentage of available milk removed and milk flow dynamics in healthy breastfeeding mothers of full-term infants during established lactation who were already experienced with breast pump use. Comfort ratings were comparable between devices, with both pumps rated as more comfortable than participants’ own home pumps. Despite the absence of functional differences, participants perceived the hospital-grade breast pump vacuum to be significantly smoother and sound more pleasant than the personal-use pump, indicating that lactating women are sensitive to subtle differences in patterns of vacuum delivery. These findings may be of practical relevance for health professionals supporting women who need to express milk for their infants and highlight the need for further investigation of devices used to establish and/or sustain lactation.

## Figures and Tables

**Table 1:** Participant characteristics.

Characteristics	Mean ± SD *n* = 28
Maternal characteristics	
Maternal age (years)	34.3 ± 4.7 ^1^
Parity	1.7 ± 0.8
Lactation stage (months)	3.5 ± 1.5
Milk production (g)	912 ± 386
Infant characteristics	
Birth gestation (weeks)	39.1 ± 2.1
Birth weight (g)	3416 ± 641

^1^ Data are mean ± standard deviation (SD).

**Table 2:** Milk removal parameters.

Parameters	HGP Mean ± SD	PUP Mean ± SD	Effect Size (95% CI)	*p*-Value 1 ^3^	*q*-Value 1 ^4^	Effect Size (95% CI)	*p*-Value 2 ^5^	*q*-Value 2 ^6^
Stimulation vacuum (mmHg)	−120.0 ± 37.2 ^1^	−114.8 ± 34.7 ^1^	−2.9 (−14.0, 8.2) ^2^	0.45	0.62	4.3 (−10.4, 19.0) ^2^	0.88	0.98
Maximum comfortable vacuum (mmHg)	−189.7 ± 53.8	−177.8 ± 52.4	−9.6 (−25.0, 6.2)	0.43	0.62	−0.4 (−19.6, 18.9)	0.31	0.82
Left breast volume (g)	75.3 ± 44.9	57.5 ± 45.8	17.7 (3.2, 32.4)	**0.019**	0.10	3.4 (−12.6, 19.3)	0.66	0.93
Right breast volume (g)	71.8 ± 43.1	67.6 ± 37.5	4.2 (−9.4, 17.9)	0.53	0.68	3.7 (−9.4, 16.9)	0.56	0.84
Combined volume (g)	147.1 ± 78.1	125.0 ± 73.5	22.0 (0.2, 43.9)	**0.048**	0.19	2.8 (−25.5, 31.1)	0.84	0.96
Post-expression cream	14.4 ± 5.1	14.9 ± 4.6	−0.1 (−2.3, 2.1)	0.91	0.94	0.7 (−1.6, 3.1)	0.52	0.84
Post-expression DOF	0.08 ± 0.10	0.11 ± 0.14	−0.004 (−0.1, 0.1)	0.91	0.94	−0.01 (−0.1, 0.1)	0.80	0.96
Left breast PAMR (%)	73.6 ± 71.2	68.4 ± 26.9	5.3 (−10.0, 20.5)	0.48	0.63	NA	NA	NA
Right breast PAMR (%)	80.9 ± 23.4	81.1 ± 28.2	−0.2 (−18.6, 18.1)	0.98	0.98	NA	NA	NA
Combined PAMR (%)	77.1 ± 20.4	74.7 ± 28.0	4.5 (−5.8, 14.8)	0.48	0.63	NA	NA	NA

^1^ Data are mean ± standard deviation (SD), ^2^ effect size and 95% confidence interval (CI). ^3^ *p*-value represents unadjusted comparison between pumps using linear mixed-effects models; ^4^ *q*-value represents unadjusted comparison between pumps after the Benjamini–Hochberg procedure. ^5^ *p*-value represents comparison between pumps using linear mixed-effects models adjusted for pre-expression degree of fullness; ^6^ *q*-value represents comparison between pumps adjusted for pre-expression degree of fullness after the Benjamini–Hochberg procedure. Bold font indicates a significant difference. DOF, degree of fullness; HGP, hospital-grade pump (Symphony (Medela AG)); NA, not applicable; PAMR, percentage of available milk removed; PUP, personal-use pump) (Pump in Style Pro (Medela AG)).

**Table 3:** Milk removal efficacy parameters.

Parameters	HGP Mean ± SD	PUP Mean ± SD	Effect Size (95% CI)	*p*-Value 1 ^3^	*q*-Value 1 ^4^	Effect Size (95% CI)	*p*-Value 2 ^5^	*q*-Value 2 ^6^
Nipple temperature change (°C)	−0.8 ± 1.6 ^1^	−1.0 ± 1.6 ^1^	−0.1 (−0.8, 0.5) ^2^	0.68	0.78	−0.04 (−0.9, 0.8) ^2^	0.92	0.99
Time to first milk ejection (min)	1.17 ± 0.61	1.63 ± 1.18	−0.5 (−1.0, 0.1)	0.081	0.27	−1.6 (−3.1, −0.1)	0.20	0.82
Overall flow duration (min)	13.4 ± 2.8	13.5 ± 2.8	−0.04 (−0.9, 0.8)	0.91	0.94	0.05 (−1.1, 1.2)	0.93	0.99
Constant flow duration (min)	11.0 ± 4.4	10.5 ± 4.1	0.9 (−1.5, 3.4)	0.43	0.62	−0.01 (−2.7, 2.6)	0.79	0.96
Milk during constant flow duration (g)	66.3 ± 41.8	51.8 ± 43.8	14.9 (−3.4, 33.1)	0.11	0.29	3.0 (−17.1, 23.0)	0.76	0.96
Constant flow rate (g/min)	7.2 ± 3.6	5.4 ± 3.0	1.9 (0.7, 3.1)	**0.004**	**0.041**	1.2 (−0.3, 2.7)	0.11	0.82
Milk removal rate (g/min)	4.9 ± 2.9	4.2 ± 2.8	0.7 (0.01, 1.4)	**0.047**	0.18	0.29 (−0.5, 1.1)	0.45	0.84
Active flow duration (min)	11.9 ± 3.3	12.0 ± 3.00	−0.2 (−1.3, 0.9)	0.70	0.79	−0.7 (−2.0, 0.6)	0.26	0.82
Active milk removal (g/min)	6.2 ± 3.1	4.8 ± 3.2	1.6 (0.4, 2.8)	**0.012**	0.075	0.7 (−0.8, 2.1)	0.33	0.84
Maximum flow rate (g/s)	0.34 ± 0.17	0.28 ± 0.16	0.1 (−0.03, 0.1)	0.20	0.44	0.1 (−0.04, 0.2)	0.24	0.82
Time to 80% milk removed (min)	7.9 ± 3.1	8.6 ± 3.1	−0.6 (−1.9, 0.7)	0.34	0.61	−0.2 (−1.9, 1.5)	0.82	0.96
Time to 90% milk removed (min)	10.2 ± 3.1	11.4 ± 3.3	−1.2 (−2.0, −0.3)	**0.012**	0.075	−0.6 (−1.7, 0.5)	0.27	0.82
Time to stop pumping (min)	12.1 ± 3.6	12.9 ± 3.6	−0.4 (−1.9, 1.1)	0.61	0.72	−0.1 (−1.7, 1.6)	0.94	0.99
Effectiveness—PAMR to time to stop (%/min)	6.7 ± 2.1	5.3 ± 3.0	1.1 (−0.6, 2.9)	0.19	0.44	1.1 (−0.8, 2.9)	0.23	0.82

^1^ Data are mean ± standard deviation (SD), ^2^ effect size and 95% confidence interval (CI). ^3^ *p*-value represents unadjusted comparison between pumps using linear mixed-effects models; ^4^ *q*-value represents unadjusted comparison between pumps after the Benjamini–Hochberg procedure. ^5^ *p*-value represents comparison between pumps using linear mixed-effects models adjusted for pre-expression degree of fullness; ^6^ *q*-value represents comparison between pumps adjusted for pre-expression degree of fullness after the Benjamini–Hochberg procedure. Bold font indicates a significant difference. HGP, hospital-grade pump (Symphony (Medela AG)); PAMR, percentage of available milk removed; PUP, personal-use pump (Pump in Style Pro (Medela AG)).

**Table 4:** Maternal perceptions of pump characteristics.

Characteristics	HGP Mean ± SD	PUP Mean ± SD	Effect Size (95% CI)	*p*-Value 1 ^3^	*q*-Value 1 ^4^	Effect Size (95% CI)	*p*-Value 2 ^5^	*q*-Value 2 ^6^
Comfort and sound perception								
Satisfied with comfort	4.2 ± 1.1 ^1^	4.4 ± 0.7 ^1^	−0.2 (−0.7, 0.2) ^2^	0.32	0.61	−0.1 (−0.7, 0.4) ^2^	0.58	0.84
Sound pleasantness	4.6 ± 0.7	3.2 ± 0.8	1.4 (1.0, 1.8)	**<0.001**	**<0.001**	1.4 (0.9, 1.8)	**<0.001**	**<0.001**
Comparison to home pump								
Comfort compared to home pump	3.5 ± 1.0	3.3 ± 0.9	0.2 (−0.3, 0.7)	0.43	0.62	0.2 (−0.4, 0.8)	0.40	0.84
Better compared to home pump	3.8 ± 0.8	3.4 ± 0.8	0.4 (0.02, 0.8)	**0.039**	0.17	0.4 (−0.1, 0.9)	0.12	0.82
Effectiveness compared to home pump	3.8 ± 0.9	3.5 ± 0.7	0.3 (−0.1, 0.7)	0.14	0.36	0.2 (−0.3, 0.6)	0.52	0.84
Satisfaction with pump								
Satisfied with milk volume	4.5 ± 0.9	4.3 ± 0.9	0.2 (−0.2, 0.6)	0.33	0.61	0.3 (0.1, 0.6)	0.43	0.84
Satisfied with vacuum strength	4.7 ± 0.6	4.5 ± 0.7	0.1 (−0.1, 0.4)	0.33	0.61	0.1 (−0.2, 0.5)	0.38	0.84
Vacuum perception								
Vacuum perceived strong and comfortable	4.4 ± 0.9	4.0 ± 0.8	0.3 (−0.1, 0.7)	0.10	0.29	0.3 (−0.2, 0.8)	0.18	0.82
Vacuum perceived smooth	4.7 ± 0.5	3.9 ± 1.4	0.9 (0.3, 1.3)	**0.003**	**0.041**	0.6 (0.1, 1.2)	**0.030**	0.57
Vacuum perceived as gentle	4.3 ± 0.8	3.9 ± 1.0	0.4 (0.1, 0.9)	0.076	0.27	0.3 (−0.2, 0.8)	0.28	0.82

^1^ Data are mean ± standard deviation (SD), ^2^ effect size and 95% confidence interval (CI). All variables were rated on a 5-point Likert scale with higher scores indicating more positive responses: sound pleasantness (1, most unpleasant to 5, most pleasant); home pump comparisons (1, much worse to 5, much better); satisfaction with pump (1, very dissatisfied to 5, very satisfied); vacuum perceptions (1, strongly disagree to 5, strongly agree). ^3^ *p*-value represents unadjusted comparison between pumps using linear mixed-effects models; ^4^ *q*-value represents unadjusted comparison between pumps after the Benjamini–Hochberg procedure. ^5^ *p*-value represents comparison between pumps using linear mixed-effects models adjusted for pre-expression degree of fullness; ^6^ *q*-value represents comparison between pumps adjusted for pre-expression degree of fullness after the Benjamini–Hochberg procedure. Bold font indicates a significant difference. HGP (hospital-grade pump; Symphony (Medela AG)); PUP (personal-use pump; Pump in Style Pro (Medela AG)).

## Data Availability

The data presented in this study are available on request from the corresponding author due to ethical restrictions.

## Abbreviations

The following abbreviations are used in this manuscript:

HGP	Hospital-grade pump
DOF	Degree of fullness
PAMR	Percentage of available milk removed
PUP	Personal-use pump
